# Prognostic value of platelet to lymphocyte ratio in non-small cell lung cancer: evidence from 3,430 patients

**DOI:** 10.1038/srep23893

**Published:** 2016-03-30

**Authors:** Xiaobin Gu, Shaoqian Sun, Xian-Shu Gao, Wei Xiong, Shangbin Qin, Xin Qi, Mingwei Ma, Xiaoying Li, Dong Zhou, Wen Wang, Hao Yu

**Affiliations:** 1 Department of Radiation Oncology, Peking University First Hospital, Beijing 100034, China; 2 Tangshan People’s Hospital, Hebei 063000, China

## Abstract

This study was designed to explore the association between elevated platelet to lymphocyte ratio (PLR) and prognosis of patients with non-small cell lung cancer (NSCLC) by meta-analysis. A total of 11 studies with 3,430 subjects were included and the combined hazard ratio (HR) and 95% confidence intervals (95% CI) were calculated. The data showed that elevated PLR predicted poor overall survival (OS) (HR = 1.42; 95% CI: 1.25–1.61, p < 0.001; I^2^ = 63.6, P_h_ = 0.002) and poor disease-free survival (DFS)/progression-free survival (PFS) (HR = 1.19; 95% CI: 1.02–1.4, p = 0.027; I^2^ = 46.8, P_h_ = 0.111). Subgroup analysis showed elevated PLR did not predict poor OS in patients included in large sample studies (HR = 1.44; 95% CI: 0.94–2.21, p = 0.098) whereas petients with Caucasian ethnicity (HR = 1.59; 95% CI: 1.27–1.98, p < 0.001) and PLR cut-off value >180 (HR = 1.61; 95% CI: 1.3–1.99, p < 0.001) had enhanced prognostic efficiency for OS. Subgroup analysis also demonstrated that high PLR did not predict poor DFS/PFS in Asian patients. In conclusion, our meta-analysis suggested that elevated PLR was associated with poor OS and DFS/PFS in NSCLC. In addition, high PLR especially predicted poor OS in Caucasians but had no association with poor DFS/PFS in Asians.

Lung cancer is one of the most commonly diagnosed cancer forms and the leading cause of cancer related mortality in both developed and developing countries^1^. Lung cancer mainly consists of non-small cell lung cancer (NSCLC) and small cell lung cancer (SCLC). NSCLC accounts for more than 80% of all lung cancer cases. The major treatment methods for NSCLC are surgery, chemotherapy and radiotherapy, in addition, targeted therapies on the specific gene mutations (e.g. EGFR, ALK etc.) have shown encouraging effects^2^,^3^. In spite of these, the 5-year survival rate of NSCLC is only 16.6% because about two-thirds of NSCLC patients are at locally advanced or metastatic stage when diagnosed^4^,^5^. The poor survival of NSCLC is partly due to absence of efficacious biomarkers. Traditional prognostic biomarkers such as ECOG PS, weight loss and pleural effusion provided limited implication for treatment and several emerging biomarkers including EGFR mutations and ALK gene rearrangements only provided useful information for clinical management for a small proportion of patients^6^,^7^. The identification of novel prognostic factors could help stratify risk patients and guide therapy modalities selection.

Accumulated evidence show that host’s inflammatory response plays an important role in cancer progression and prognosis^8^,^9^. In recent years, a variety of inflammatory indices such as neutrophil to lymphocyte ratio (NLR), platelet to lymphocyte ratio (PLR), C-reactive protein (CRP) and modified Glasgow prognostic score (mGPS) have attracted extensive attention for their prognostic efficiency in cancer patients^10^,^11^. Notably, as an easily measured blood-based parameter, PLR was reported as an unfavourable prognostic factor in various solid tumors including gastric cancer^12^, breast cancer^13^, colorectal cancer^14^ and NSCLC^15^. However, the data concerning the prognostic value of PLR in NSCLC were inconsistent. Liu *et al.*^15^ reported that elevated PLR was associated with poor prognosis in patients with NSCLC receiving chemotherapy.

Cannon *et al.*^16^ also showed that patients with high pretreatment PLR had shorter overall survival after stereotactic radiation therapy. In contrast, Pinato *et al.*^17^ failed to find the prognostic significance of PLR in primary operable NSCLC and Wu *et al.*^18^ also did not find correlation between PLR and prognosis of NSCLC. We thus collected the available publications and conducted this meta-analysis to disclose the prognostic role of PLR for overall survival (OS), disease-free survival (DFS)/progress-free survival (PFS) in NSCLC.

## Results

### The characteristics of included studies

**Figure 1:**
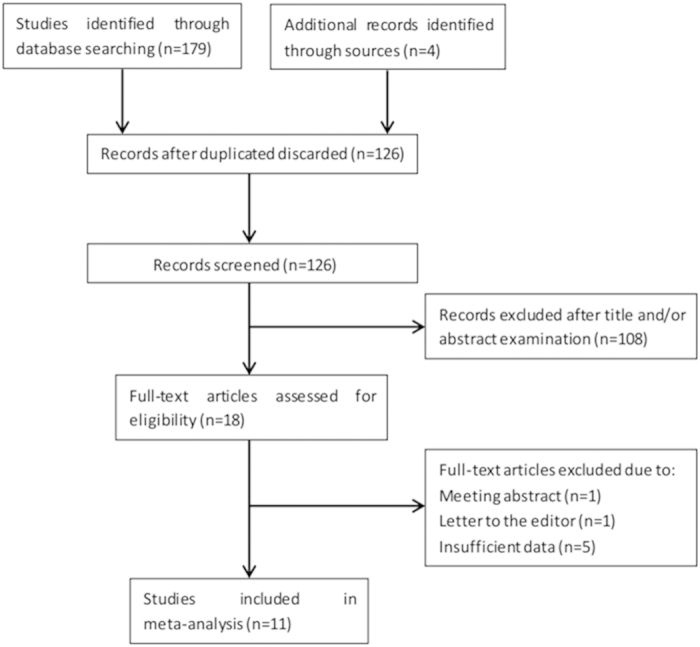
Flow chart of the study selection.

The literature selection process of the eligible studies was presented in Fig. 1. A total of 11 studies^15^,^16^,^17^,^18^,^19^,^20^,^21^,^22^,^23^,^24^,^25^ with 3,430 patients were included in the meta-analysis. The basic characteristics of the primary studies were shown in Table 1. Of these studies, four studies^15^,^18^,^20^,^24^ were conducted in China, two studies^16^,^23^ were conducted in USA, two studies^19^,^22^ were performed in Turkey, two studies^21^,^25^ were carried out in Japan and one study^17^ was conducted in UK. Three studies^15^,^18^,^19^ included patients with advanced tumor stages, seven studies^16^,^17^,^20^,^21^,^23^,^24^,^25^ involved patients with early stages and one study^22^ included patients with all tumor stages. The articles were published from 2013 to 2015 and the NOS scores of the included studies ranged from 7–9. Five studies^16^,^17^,^18^,^21^,^24^ gave the definition of OS and defined OS as the length of time from treatment to day of death or last follow-up. The other six studies^15^,^19^,^20^,^22^,^23^,^25^ generally described OS as overall survival. One study^24^ provided the definition of DFS and defined DFS as theduration of time between the date of treatment and the date of first recurrence or last follow-up. Three studies^19^,^20^,^25^ generally described DFS as disease-free survival. One study^18^ defined PFS as the time from treatment initiation until disease progression. All the 11 studies^15^,^16^,^17^,^18^,^19^,^20^,^21^,^22^,^23^,^24^,^25^ investigated the prognostic value of PLR in OS and five studies^18^,^19^,^20^,^24^,^25^ explored the prognostic significance of PLR in DFS/PFS. The sample sizes in the included syudies ranged from 59 to 1043. Four studies^18^,^20^,^21^,^24^ were classified as large sample size (n > 300) studies and seven studies^15^,^16^,^17^,^19^,^22^,^23^,^25^ were small sample size (n < 300) studies. The cut-off values used by the included studies ranged from 106 to 300, the median value of which was 171, so we selected PLR = 180 to divide the cut-off values in the following subgroup analysis.

### PLR and OS in NSCLC

**Figure 2:**
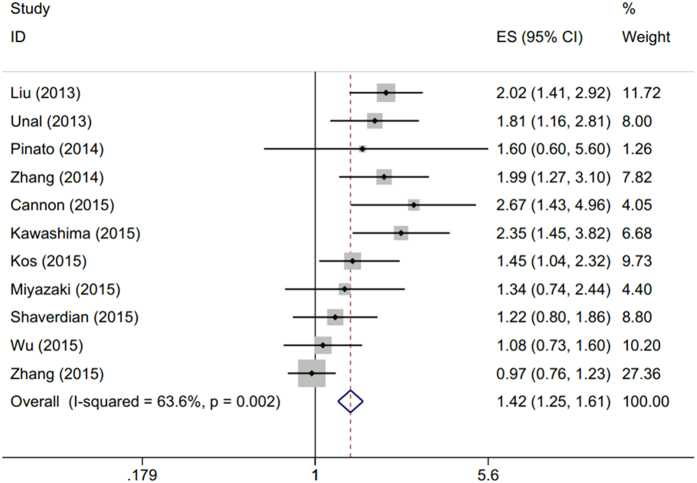
Forest plot of the association between PLR and OS in patients with NSCLC.

Eleven studies^15^,^16^,^17^,^18^,^19^,^20^,^21^,^22^,^23^,^24^,^25^ with 3,430 patients reported the data of pretreatment PLR and OS in NSCLC. Elevated PLR was associated with poor OS (HR = 1.42; 95% CI: 1.25–1.61, p < 0.001) although with heterogeneity (I^2^ = 63.6, P_h_ = 0.002; Table 2, Fig. 2).

### PLR and DFS/PFS in NSCLC

**Figure 3:**
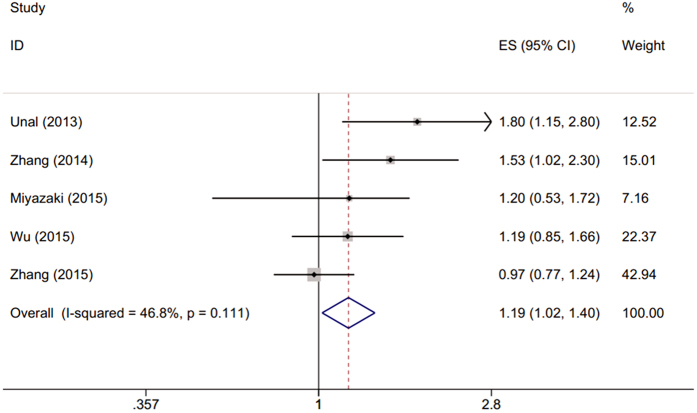
Forest plot of the association between PLR and DFS/PFS in patients with NSCLC.

There were five studies^18^,^19^,^20^,^24^,^25^ with 1,635 patients presenting the HR and 95% CI of PLR and DFS/PFS. The combined data showed that elevated PLR was associated with shorter DFS/PFS (HR = 1.19; 95% CI: 1.02–1.4, p = 0.027) with moderate heterogeneity (I^2^ = 46.8, P_h_ = 0.111; Table 2, Fig. 3).

### Subgroup analyses

To detect the potential source of heterogeneity, subgroup analyses stratified by ethnicity, sample size, treatment methods and PLR cut-off were performed. As shown in Table 2, elevated PLR did not predict poor OS in patients in large sample studies (HR = 1.44; 95% CI: 0.94–2.21, p = 0.098; I^2^ = 80.8, P_h_ = 0.001), however, elevated PLR had enhanced prognostic efficiency for poor OS in Caucasians (HR = 1.59; 95% CI: 1.27–1.98, p < 0.001; I^2^ = 15.2, P_h_ = 0.318) and when the cut-off value of PLR was more than 180 (HR = 1.61; 95% CI: 1.3–1.99, p < 0.001; I^2^ = 11.8, P_h_ = 0.339). As for the PLR in DFS/PFS, the results showed that elevated PLR did not predict poor DFS/PFS in Asians (HR = 1.12; 95% CI: 0.94–1.34, p = 0.205; I^2^ = 46.6 P_h_ = 0.154) whereas high PLR was correlated with shortened DFS/PFS in small sample studies(HR = 1.55; 95% CI: 1.09–2.22, p = 0.015; I^2^ = 13.8,P_h_ = 0.281) (Table 2).

### Sensitivity analysis

**Figure 4:**
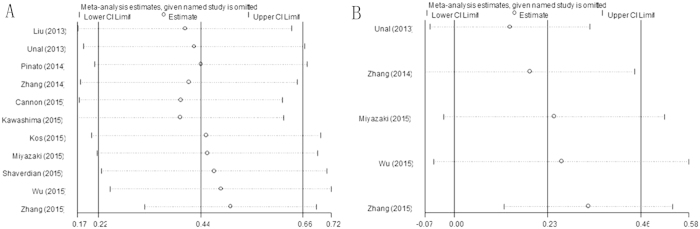
Sensitivity analysis on the relationship between PLR and (**A**) OS and (**B**) DFS/PFS in NSCLC.

Each single study was omitted every time to estimate the influence of individual data sets on the combined HR. The results showed that the pooled HRs for OS and DFS/PFS were not substantially changed (Fig. 4), indicating the robustness of our findings.

### Publication bias

**Figure 5:**
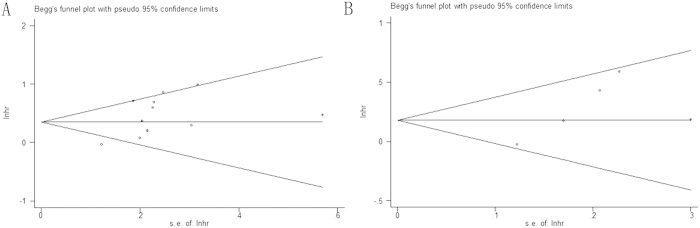
Begg’s funnel plot of publication bias test for (**A**) OS and (**B**) DFS/PFS in NSCLC.

Begg’s test suggested no evidence of obvious publication bias (p = 0.119 for OS and p = 0.221 for DFS/PFS, respectively)(Fig. 5).

## Discussion

In the present study, using the method of meta-analysis, we explored the prognostic impact of pretreatment PLR on OS and DFS/PFS in patients with NSCLC. By combining the HRs and 95% CIs from eleven primary studies^15^,^16^,^17^,^18^,^19^,^20^,^21^,^22^,^23^,^24^,^25^ with 3,430 subjects, we showed that elevated PLR was associated with poor OS (HR = 1.42; 95% CI: 1.25–1.61, p < 0.001) and poor DFS/PFS (HR = 1.19; 95% CI: 1.02–1.4, p = 0.027) in NSCLC. Furthermore, stratified analysis showed that high PLR had consistent prognostic value in NSCLC in diverse subgroup populations expect for patients included in large sample size studies (HR = 1.44; 95% CI: 0.94–2.21, p = 0.098), whereas patients with Caucasuian ethnicitic background and PLR > 180 could better predicted poor OS. The stratified analysis also showed that high PLR had no prognostic efficiency for DFS/PFS in Asian patients. All of the studies were published since 2013, highlighting the recent interest in PLR as an attractive prognostic factor. To our knowledge, this was the first meta-analysis to investigate the association between PLR and NSCLC prognostication.

Inflammation and immune responses were recognized as important stimulators for tumorigenesis since it was first proposed by Virchow^26^ in the 19th century. In the past several decades, a large amount of studies investigating mechanisms by which inflammation promote tumorigenesis suggested that inflammatory cells are important cross-talk factors between chronic inflammation and neoplastic growth^27^. Lung cancer patients often have the common feature of chronic inflammation, such as COPD^27^,^28^. In the tumor microenvironment, macrophages, neutrophils, platelets and lymphocytes produce inflammatory cytokines and chemokines, which could facilitate tumor progression^29^. A variety of cytokine proteins such as IL-1, IL-6 and TNF could enhance tumor cells’ capability to metastasize^30^. Leukocyte infiltration was also shown to be related with tumor angiogenesis^31^. Readily available blood based parameters including NLR, PLR and mGPS could adequately reflect the cancer-related inflammatory status and are widely investigated as prognostic factors in NSCLC^32^,^33^.

The results of this meta-analysis provided evidence supporting elevated PLR as a prognostic factor for OS in NSCLC, which was in line with a previous meta-analysis^34^. In addition, we noticed that in the previous work^34^, a variety of solid tumors were included for analysis, except for NSCLC. The previous meta-analysis^34^ searched literature until June 2013, but the first eligible primary study^15^ included in our meta-analysis was published on December 2013. Therefore, the current study first provided the statistical evidence for PLR’s prognostic role in NSCLC by meta-analysis. Interestingly, after subgroup analysis dichotomized by sample sizes of included studies, we found that high PLR no longer predicted poor OS in patients attending large sample size studies. (Table 2). However, four studies^18^,^20^,^21^,^24^ with 2,487 patients were identified as large sample studies, one^21^ of which recruited 1,043 subjects. This study^21^ may have significant impact on the results of subgroup analysis stratified by sample size, therefore, the results should be interpreted with caution. Furthermore, subgroup analysis demonstrated that patients with Caucasian ethnicitic background and a higher PLR (>180) had augmented prognostic value, because a higher PLR represented more seriously impaired immune functions in cancer patients. The prognostic role of PLR for DFS/PFS was also detected in our study whereas elevated PLR did not suggest poor DFS/PFS in Asian patients in subgroup analysis. The ethnicitic heterogenicity may account for the results. Neutrophil-to-lymphocyte ratio (NLR) was another easily available and useful index for prognosis prediction in NSCLC. Our previous work^35^ had demonstrated NLR might be a predicative factor of poor prognosis for NSCLC patients. In the current study, we intended to explore the prognostic role of PLR in NSCLC, which was usually compared with NLR in prognostication. We pooled coflicting data from 11 studies and showed the prognostic value of PLR for NSCLC, which extended the inflammatory prognostic factors for NSCLC.

The present study had several limitations. First, obvious heterogeneity existed in this meta-analysis. Although sensitivity analysis and publication bias test indicated the credibility of the results, we could not rule out that different study criteria used in the primary resulted in the discrepancies between studies. Second, the nonuniform cutoff value defining elevated PLR may not be applicable for clinical use, an identical cutoff value was needed. Finally, the summary HR and 95% CI rather than individual patient data were used for calculation of pooled HR and 95% CI in this meta-analysis.

In conclusion, our study for the first demonstrated the prognostic role of elevated PLR for poor OS and DFS/PFS in NSCLC by meta-analysis. Considering the limitations of our study, further well-designed studies using uniform PLR cutoff value are warranted to test our results.

## Methods

### Search strategy and eligibility criteria

The databases of Web of Science, Embase and Pubmed were thoroughly searched until December, 2015. The following terms were used in separation or in combination: “PLR”, “platelet-lymphocyte ratio”, “platelet to lymphocyte ratio”, “lung cancer”, “lung carcinoma” or “NSCLC”. Reviews and reference lists were also manually retrieved for additional publications. The publication language was limited to English.

The inclusion criteria were: 1) patients pathologically diagnosed as NSCLC; 2) PLR was measured by blood-based methods before formal treatment; 3) HRs and 95% CIs for PLR in OS and (or) DFS/PFS were reported in text or sufficient data was provided for the calculation of HRs and 95% CIs. 4) full text papers published in English.

The exclusion criteria were as follows: 1) review, meeting abstract, letter, not full text in English; 2) duplicate data; 3) nonhuman studies; 4) did not present the cut-off value for elevated PLR.

### Data extraction

Two independent reviewers (XB,G and XS,G) extracted the following information from the eligible studies: the surname of the first author, year of publication, study country, sample size, treatment methods, cut-off value of high PLR and survival data. Disagreements were resolved by joint discussion.

### Quality assessment

The quality assessment of primary studies was performed according to Newcastle-Ottawa quality assessment Scale (NOS)(http://www.ohri.ca/programs/clinical_epidemiology/oxford.asp). This scale is composed of three parts: selection, comparability and outcome assessment. The full mark is 9 points and studies labeled with ≥6 points were regarded as high-quality researches.

### Statistical analysis

The hazard ratio (HR) with 95% confidence intervals(95% CI) were directly obtained from the articles or estimated according to the methods reported by Tierney *et al.*^36^. Heterogeneity among primary studies was evaluated using the Cochran Q test and I^2^ statistic. Cochran Q test’p value < 0.10 or I^2^ > 50% indicated large heterogeneity between studies and random effects models (DerSimonian Laird method) was used to calculate the pooled HR and 95% CI. Otherwise, the fixed effects model (Mantel-Haenszel method) was used. Studies with sample size >200 were considered as large sample studies, otherwise was regarded as small sample size. Subgroup analyses stratified by ethnicity, sample size, treatment methods and PLR cut-off were carried out.

Sensitivity analysis was conducted by omitting each single study and recalculating their HRs. Publication bias was evaluated using Begg’s test^37^. All statistical analyses were performed using Stata 12(Stata Corp., College Station, Texas). P < 0.05 was considered statistically significant.

## Additional Information

**How to cite this article**: Gu, X. *et al.* Prognostic value of platelet to lymphocyte ratio in non-small cell lung cancer: evidence from 3,430 patients. *Sci. Rep.* **6**, 23893; doi: 10.1038/srep23893 (2016).

## Figures and Tables

**Table 1:** Characteristics of all included studies.

Study	Year	Country	Ethnicity	Follow-up (month)	Sample size	Gender (M/F)	TNM stage	Cut-off	Treatment methods	Outcome	Hazard ratio	Study design	NOS score
Liu^15^	2013	China	Asian	To Aug 2012	210	139/71	III–IV	152.6	Chemotherapy	OS	R	Retrospective	8
Unal^19^	2013	Turkey	Caucasian	NA	94	88/6	II–IIIB	194	Chemoradiotherapy	OS,DFS	R	Retrospective	8
Pinato^17^	2014	UK	Caucasian	To Sep 2012	220	110/110	I–III	300	Surgery	OS	R	Prospective	7
Zhang^20^	2014	China	Asian	46(1–78)	400	272/128	I–II	171	Surgery	OS,DFS	R	Retrospective	8
Cannon^16^	2015	USA	Caucasian	17(median)	59	31/28	I	146	Radiotherapy	OS	E	Retrospective	7
Kawashima^21^	2015	Japan	Asian	NA	1043	671/372	I–III	300	Surgery	OS	R	Retrospective	7
Kos^22^	2015	Turkey	Caucasian	33(1–128)	145	130/15	I–IV	198.2	Mixed	OS	R	Retrospective	9
Miyazaki^25^	2015	Japan	Asian	NA	97	62/35	I	118	Surgery	OS,DFS	E	Retrospective	8
Shaverdian^23^	2015	USA	Caucasian	28.9(median)	118	NA	I–II	187.27	Radiotherapy	OS	E	Retrospective	7
Wu^18^	2015	China	Asian	To Dec 2013	366	246/120	III–IV	119.5	Chemotherapy	OS,PFS	R	Retrospective	7
Zhang^24^	2015	China	Asian	43.5(1–99)	678	449/229	I–III	106	Surgery	OS,DFS	R	Retrospective	7

NA: not available; R: reported in text; E: estimated; OS: overall survival; DFS: disease free survival; PFS: progressi on free survival; NOS: Newcastle–Ottawa Quality Assessment Scale.

**Table 2:** Summary of the meta-analysis results.

	Variable	No. of studies	No. of patients	Effects model	HR (95% CI)	p	Heterogeneity	
							I^2^(%)	P_h_
OS	Overall	11	3,430	R	1.42(1.25–1.61)	<0.001	63.6	0.002
	Ethnicity							
	Asian	6	2,794	R	1.51(1.08–2.11)	0.016	76.6	0.001
	Caucasian	5	636	F	1.59(1.27–1.98)	<0.001	15.2	0.318
	Sample size							
	Large	4	2,487	R	1.44(0.94–2.21)	0.098	80.8	0.001
	Small	7	943	F	1.66(1.38–1.99)	<0.001	7.8	0.369
	Treatment							
	Nonsurgery	6	992	R	1.58(1.23–2.02)	<0.001	50.4	0.073
	Surgery	5	2,438	R	1.54(1–2.35)	0.048	73.7	0.004
	Cut-off							
	PLR < 180	6	1,810	R	1.52(1.08–2.14)	0.017	76.1	0.001
	PLR > 180	5	1,620	F	1.61(1.3–1.99)	<0.001	11.8	0.339
DFS/PFS	Overall	5	1,635	F	1.19(1.02–1.4)	0.027	46.8	0.111
	Ethnicity							
	Asian	4	1,541	R	1.13(0.98–1.33)	0.165	20.8	0.285
	Caucasian	1	94	−	1.8(1.15–2.81)	0.01	−	−
	Sample size							
	Large	3	1,444	F	1.12(0.94–1.34)	0.205	46.6	0.154
	Small	2	191	F	1.55(1.09–2.22)	0.015	13.8	0.281
	Treatment							
	Nonsurgery	2	460	R	1.42(0.95–2.13)	0.086	53.3	0.144
	Surgery	3	1,175	F	1.11(0.91–1.34)	0.312	45.3	0.161

R: random-effects model; F: fixed-effects model; P_h_: p value of Q test for heterogeneity.
